# Post-exposure prophylaxis for sexual assault victim-survivors: Guidelines and best practices

**DOI:** 10.4102/safp.v67i1.6073

**Published:** 2025-06-05

**Authors:** Mergan Naidoo, Ramprakash Kaswa, Indiran Govender

**Affiliations:** 1 Department of Family Medicine, College of Health Sciences, University of KwaZulu-Natal, Durban, South Africa; 2 Department of Family Medicine and Rural Health, Faculty of Health Sciences, Walter Sisulu University, Mthatha, South Africa

**Keywords:** sexual assault, post-exposure prophylaxis, national guidelines, victim-survivors

## Abstract

This study addresses the importance of post-exposure prophylaxis (PEP) in the context of sexual assault. Post-exposure prophylaxis serves as a critical intervention to reduce the risk of human immunodeficiency virus (HIV) transmission and unintended pregnancies for victim-survivors. Immediate access to PEP, emergency contraception and comprehensive medical assessments is essential for effective care. The study outlines the steps healthcare providers must take, including timely administration of prophylaxis, monitoring for side effects and offering psychosocial support to victim-survivors. It emphasises the need for follow-up visits to ensure ongoing care and the importance of implementing risk-reduction strategies until final infection outcomes are confirmed. Additionally, the role of standardised documentation, such as the J88 form, is highlighted for collecting evidence in cases of sexual violence, ensuring that healthcare practitioners understand their responsibilities in promoting justice. The study underscores the social obligation of healthcare professionals to combat gender-based violence, advocating for reporting mechanisms for child victims and appropriate referral pathways for positive test results. By prioritising the health and wellbeing of victim-survivors, the healthcare community can significantly contribute to their recovery and empowerment, ultimately fostering a supportive environment that addresses both medical and emotional needs following sexual assault.

## Introduction

South Africa faces one of the highest rates of sexual violence globally, putting victim-survivors at elevated risk of acquiring human immunodeficiency virus (HIV) and other sexually transmitted infections (STIs). Among various modes of HIV exposure, sexual assault ranks among the highest, particularly in cases of receptive anal intercourse, which carries an estimated transmission rate of 138 per 10 000 exposures, followed by needle-sharing, percutaneous injury and receptive vaginal intercourse.^1^ However, despite the urgency for post-exposure prophylaxis (PEP) to prevent HIV and other infections, many victim-survivors do not receive PEP interventions within the prescribed time.

While significant progress has been made in HIV treatment, the disease remains a leading public health burden in South Africa, home to the largest HIV-positive population globally. As reported in the Joint United Nations Programme on HIV/Acquired Immunodeficiency Syndrome (AIDS) (UNAIDS) 2023 data, an estimated 8 million South Africans live with HIV, with an incidence rate of 7 per 1000 individuals aged 15–49.^2^ Human immunodeficiency virus disproportionately affects young women and girls, who make up a considerable portion of new infections, often because of exposure to sexual violence or high-risk environments.^3,4^ Despite the availability of preventive care like PEP, many cases of sexual assault are underreported, and access to timely intervention remains limited.

The public health implications of sexual assault extend beyond HIV to hepatitis B (HBV), with South Africa facing a chronic HBV infection rate of around 6.5% despite the availability of an HBV vaccine. In response to these overlapping epidemics, South Africa implemented the PEP programme in 2002, initially distinguishing between occupational and non-occupational exposure but later adopting a standardised triple antiretroviral therapy (ART) regimen for all cases in the 2015 guidelines and updated in 2021.^5^ Effective PEP treatment requires initiation within 72 h, adherence and course completion, yet stigma, psychological trauma and limited multidisciplinary support often hinder these factors among sexual assault victim-survivors.^5^

The client’s health needs are the most important factor that needs to be considered post-sexual assault. It includes the treatment and prevention of HIV, hepatitis B and hepatitis C, as well as pregnancy prevention, STI and psychological support.^3,6^ The effectiveness of PEP depends on the level of adherence and course completion. In addition, other factors such as treatment timing, exposure risk level and the possible emergence of drug resistance can also affect its effectiveness. Because of these, it is not 100% effective and should only be considered as part of a broader strategy to prevent acquiring HIV and other blood-borne viruses.^6^

The Thuthuzela Care Centers (TCC) were introduced in 2006 to provide comprehensive care and support for victim-survivors of sexual assault by reducing re-victimisation and aiding in the legal process.^7^ The TCCs is a one-stop shop to address victim-survivors’ immediate and long-term health needs, including HIV and HBV prevention, psychological support and STI and pregnancy prevention.^7^ Post-exposure prophylaxis for sexual assault in South Africa highlights the complexity of care, where healthcare practitioners must navigate the intersection of the medical, legal and psychological needs of victim-survivors. This includes ensuring timely administration of PEP, managing injuries and addressing trauma, all the while adhering to legal requirements such as collecting forensic evidence and facilitating reporting processes. Challenges such as limited training, stigma and inadequate resources further complicate effective intervention. Disparities in access to care compound these issues, with urban centres often having better-equipped facilities and more trained personnel compared to rural areas, where healthcare infrastructure and availability of PEP may be limited. These disparities can result in delayed treatment, increased risk of HIV transmission and uneven support for victim-survivors, underscoring the urgent need for equitable healthcare systems and integrated care pathways.^8^

This guideline seeks to integrate the national and provincial guidelines. It underscores the importance of following updated PEP guidelines for healthcare providers across South Africa, ensuring effective management and support for sexual assault victim-survivors at risk of HIV and other STIs.

## Approach to a sexual assault case

### Initial assessment and medical emergency management

Healthcare providers must thoroughly evaluate anyone who may have been exposed to potentially infectious substances. This involves carefully evaluating any wounds and understanding the circumstances of the exposure. Providing immediate first aid is essential. For patients who have experienced sexual assault within the past 72 h, urgent medical care and access to PEP are critical. Every effort should be made to ensure they receive this care before leaving the facility. Where feasible, comprehensive investigations should be conducted on both the source of the potential infection and the exposed individual to identify any co-occurring infections. If the source is either unavailable or unwilling to be tested, they should be presumed HIV-positive for care and preventive measures.^5^

### Establish post-exposure prophylaxis eligibility

Post-exposure prophylaxis eligibility depends on the risks of exposure, the client’s medical history and considerations related to pregnancy or childbearing potential. Additional factors include the time elapsed since exposure and the possibility of multiple injuries. For instance, individuals who have experienced sexual assault may also have wounds that necessitate tetanus prophylaxis.^3,5^

Laboratory and clinical assessments of both the exposed individual and the source are essential for identifying the risks of exposure. However, it is crucial not to delay initiating PEP while awaiting confirmatory test results for either the source or the exposed individual (Table 1).^5^

**TABLE 1:** Guideline for the investigations.

Investigation	Baseline	6th week	12th week
**Investigation for the exposed**			
HIV	HIV ELISA If < 2 years HIV PCR**	HIV ELISA If < 2 years HIV PCR	HIV ELISA If < 2 years HIV PCR
Hepatitis B	HBsAg	-	HBsAg
Hepatitis C	HCV Ab	HCV PCR	-
Syphilis	TP Ab/RPR	-	TP Ab/RPR
Creatinine	If TDF is part of PEP	-	-
Hb	If AZT is part of PEP	-	-
Pregnancy	βHCG	βHCG	
**Investigation on source**			
HIV	HIV ELISA HIV viral load	-	-
Hepatitis B	HBsAg	-	-
Hepatitis C	HCV Ab	-	-
Syphilis	TP Ab/RPR	-	-

*Source:* South African National Department of Health. National clinical guidelines of Post-Exposure Prophylaxis (PEP) in occupational and non-occupational exposures 2020. Pretoria; 2021

ELIZA, enzyme-linked immunosorbent assay; PCR, polymerase chain reaction; HBsAg, hepatitis B surface antigen; HCV Ab, hepatitis C antibodies; TP Ab, *Treponenam pallidum* antibodies; RPR, rapid plasma reagent; TDF, tenofovir; AZT, zidovudine; ΒHCG, beta human chorionic gonadotropin.

### Counselling and support

Counselling the exposed individual on the nature of the exposure and potential health risks is essential. Post-exposure prophylaxis must be started as promptly as possible, ideally within the recommended timeframes identified during the initial assessment. Counselling and support systems are crucial for adherence and completing the full course of PEP.

For HIV prevention, individuals must prioritise follow-up appointments and testing. Regular counselling and support can help reinforce this commitment. Beyond discussing health conditions, emotional support should also be provided. If the individual is unable to manage their needs because of emotional distress or injury, assistance and counselling should be offered. Post-exposure prophylaxis initiation should not be delayed, and ongoing counselling should be readily available to address any potential medication side effects.^5,9^

### Provide immediate human immunodeficiency virus post-exposure prophylaxis

Post-exposure prophylaxis should be initiated immediately, even if confirmatory test results are not yet available or healthcare providers are uncertain of the potential risk of HIV exposure. It is essential to provide counselling based on the patient’s understanding of the benefits and risks of PEP. Prior to prescribing, providers must also assess potential side effects and drug interactions.

A full 28-day supply of medication for HIV PEP should be provided, as starter packs may lead to non-adherence. These updated guidelines aim to simplify the prescribing process. One of the most common reasons for discontinuation of medication is the experience of side effects; therefore, selecting appropriate regimens and managing side effects is critical for the success of PEP (Table 2).^5^

**TABLE 2:** Preferred post-exposure prophylaxis regimens.

Clinical scenario	Therapeutic option
**Individuals 10 years and older with a weight of at least 30 kg**	
Uncomplicated patient	TDF (300 mg) + 3TC (300 mg) + DTG (50 mg) fixed-dose combination tablet (TLD) once daily
If DTG is intolerable	TDF (300 mg) + FTC (200 mg) + ATV/r (300/100 mg) as once daily dose or TDF (300 mg) once daily + FTC (200 mg) once daily + LPV/r (200/50 mg) two tablets twice daily
If renal failure	If eGFR 10 mL/min – 50 mL/min: AZT 300 mg bd + 3TC 150 mg daily + DTG 50 mg daily If eGFR < 10 mL/min: AZT 300 mg daily + 3TC 50 mg daily + DTG 50 mg daily
**Individuals less than 10 years old or less than 30 kg**	
First option	AZT + 3TC + DTG
If DTG is intolerable or not available	AZT + 3TC + protease inhibitor (ATV/r or LPV/r)

*Source:* South African National Department of Health. National clinical guidelines of Post-Exposure Prophylaxis (PEP) in occupational and non-occupational exposures 2020. Pretoria; 2021

3TC, lamivudine; DTG, dolutegravir; FTC, emtricitabine; ATVr, atazanavir/ ritonavir; LPV/r, lopinavir/ritonavir; eGFR, estimated glomerular filtration rate.

### Pregnancy test and provide emergency contraception

Emergency contraception pills should be taken as soon as possible, ideally within 5 days of exposure. They can be used at any time during the menstrual cycle. The primary focus of care is to determine whether the individual has already become pregnant before the sexual encounter while also respecting a woman’s autonomy to decide whether she wishes to prevent pregnancy following sexual assault.^3^

In South Africa, there are two types of emergency contraceptives available: hormonal emergency contraceptive pills and the copper intrauterine device (IUD), both of which can be used up to 120 h after unprotected sexual intercourse, although sooner is preferable. Levonorgestrel is the preferred regimen and is less invasive. Enzyme inducers (including efavirenz and carbamazepine) cause a significant reduction in levonorgestrel concentrations. Women on these medicines should double the dose of levonorgestrel because of the significant reduction of levonorgestrel. Women with a weight of 80 kg or more or body mass index of ≥ 30 should also be given twice the standard dose.^5^

### Screen for and manage sexually transmitted infections

If an individual has been sexually exposed to other STIs, they need to undergo a thorough examination at the time of exposure. A follow-up visit should also be scheduled to allow sufficient time to develop antibodies. Blood tests for syphilis should be conducted 12 weeks after exposure. In accordance with the National Department of Health (NDOH) guidelines and the national sexual assault policy, pre-treatment for STIs is provided during the initial visit (Table 3).^3,9^

**TABLE 3:** Sexually transmitted infection drug prophylaxis.

Drug	Dosage	Route	Frequency
Ceftriaxone	250 mg	Intramuscularly (IM)	Single dose
Azithromycin	1 g	Orally	Single dose
Metronidazole	2 g	Orally	Single dose

*Source:* South African National Department of Health. National clinical guidelines of Post-Exposure Prophylaxis (PEP) in occupational and non-occupational exposures 2020. Pretoria; 2021

### Screen and prevent hepatitis B transmission

As the likelihood of acquiring hepatitis B is generally higher than that of HIV in most cases, it is essential to manage all exposures to hepatitis B infection. The effectiveness of hepatitis B prophylaxis is primarily determined by the timing of the first dose of the HBV vaccine and hepatitis B immune globulin (HBIG). These should be administered within 24–72 h following exposure. However, the effectiveness of the vaccine diminishes with increasing delays and is unlikely to be effective after 14 days post-exposure.^3,6^

For individuals who have previously been vaccinated against hepatitis B, it is important to receive the next dose according to the immunisation schedule. The concurrent use of passive immunisation with hepatitis B immunoglobulin (HBIG) can also protect against hepatitis B virus infection following exposure (Table 4).^3^

**TABLE 4:** Hepatitis B infection risk management.

Hepatitis B status	Vaccination status and antibody response of the exposed person	Treatment
Hepatitis B status of the source	Not vaccinated Unsure if vaccinated Vaccination incomplete Vaccinated but with HBsAb < 10 IU/mL, or level unknown	Vaccinated with known response i.e. HBsAb > 10 IU/mL
HBsAg positive or unknown	HBIG, IM, 500 units Hep B vaccine (3 doses at monthly intervals)	No treatment
HBsAg negative	Initiate/complete/repeat HBV vaccination (months 0, 1 and 6)	No treatment

*Source:* South African National Department of Health. National clinical guidelines of Post-Exposure Prophylaxis (PEP) in occupational and non-occupational exposures 2020. Pretoria; 2021

HBsAb, hepatitis B surface antibody.

### Screen and prevent hepatitis C transmission

Currently, there is no vaccine for the hepatitis C virus (HCV). If the source is tested negative for the virus, the exposed person does not need any follow-up test. However, if the source is positive, the exposed person should be tested for HCV antibodies at the beginning of the exposure and then again 6 weeks later for an HCV PCR test.^5^

### Prevention of tetanus

The bacterium known as *Clostridium tetani* can induce tetanus, a fatal illness. It can enter the body through an open wound or puncture. To prevent the spread of the infection, people must get vaccinated and clean their wounds. Regular boosters are needed to maintain immunity. Those with cuts, abrasions or bites and those in which objects have been used should be immunised for tetanus if not immunised or if they have not received a booster in the previous 5 years (Table 5).^5,6^

**TABLE 5:** Tetanus risk management.

Immunisation status	Clean, minor wound		All other wounds	
	TT, IM 0.5 mL	TIG	TT, IM 0.5 mL	TIG
Not immunised in the last 5 years	Yes	No	Yes	Yes
Immunised in last 5 years	No	No	No	No

*Source:* South African National Department of Health. National clinical guidelines of Post-Exposure Prophylaxis (PEP) in occupational and non-occupational exposures 2020. Pretoria; 2021

TT, tetanus toxoid; TIG, tetanus immunoglobulin.

## Forensic specimen and J88 form for gender-based violence reporting

The J88 form, produced by the Department of Justice, serves as the standard document for collecting evidence in criminal cases. It is specifically designed to gather information regarding injuries sustained by complainants in instances of rape, physical assault and sexual assault. All healthcare practitioners involved in such cases must be aware of their roles and responsibilities. As medical professionals, they must ensure that justice is served in cases of rape and assault.

In addition to their responsibility towards patients, healthcare professionals have a social obligation to combat gender-based violence. Furthermore, all documentation related to the incident should be meticulously recorded on the J88 form, alongside supported forensic sample collection.^5,7^ Mandatory reporting scenarios include the following.

### Sexual assault on a child

Under the *Children’s Act* (No. 38 of 2005), healthcare professionals are legally obligated to report to the police or social services if they have reasonable grounds to believe that a child (under 18) has been sexually assaulted or is at risk of sexual harm.^10^

### Sexual assault on a vulnerable person

The Criminal Law (Sexual Offences and Related Matters) Amendment Act (No. 32 of 2007) mandates reporting if the victim is mentally or physically disabled and unable to protect themselves from harm. Children under the age of 16 are considered as statutory rape unless the perpetrator has an age gap of two or less years.^11^

### Serious bodily harm

In cases where sexual assault causes severe physical injury, doctors may be required to report the incident, particularly if there is a clear risk to public safety or the victim’s wellbeing.^11^

### Post-care strategies

Post-care strategies for victim-survivors of sexual assault in South Africa must extend beyond individual care to encompass comprehensive follow-up and education efforts. Effective follow-up involves ensuring adherence to PEP regimens, managing ongoing physical and psychological health and providing long-term support to mitigate trauma. Innovative approaches, such as the use of technology and telemedicine, offer promising avenues to address barriers like geographical inaccessibility and limited resources in rural areas. Telemedicine platforms can facilitate virtual counselling, PEP adherence monitoring and consultations, reducing the need for victim-survivors to travel to healthcare facilities. Educational programmes, delivered via mobile applications or community outreach, can empower victim-survivors and communities with knowledge about available resources, prevention of sexual violence and destigmatisation. Integrating these strategies into existing care models, such as South Africa’s TCCs, could significantly enhance the continuity of care and improve outcomes for victim-survivors.^5,7^

## Follow-up visits

Follow-up visits are essential to ensure that all aspects of treatment are addressed effectively. If the prophylactic regimen is still in progress, it is essential to monitor for side effects and reassess potential drug interactions. Additionally, healthcare providers should ensure that the patient receives the necessary psychosocial support.

Support is crucial for individuals who have experienced sexual assault, and it is vital to emphasise the importance of implementing risk-reduction strategies until the final infection outcomes are confirmed. Relevant follow-up laboratory tests for any potential exposures should also be conducted as indicated. In the event of a positive result, the healthcare team should facilitate the patient’s connection to the appropriate care and treatment services they need.^5^

## Legal and ethical considerations

Providing care to victim-survivors of sexual assault in South Africa involves navigating complex ethical considerations. Central to this is obtaining informed consent for medical examinations, PEP and the collection of forensic evidence. Victim-survivors must have the autonomy to make decisions about their care without coercion, particularly in a context where trauma may impair their ability to provide immediate consent. Mandatory reporting laws introduce further ethical challenges, requiring healthcare providers to balance legal obligations with the victim-survivor’s right to confidentiality and self-determination. Additionally, care providers must navigate the tension between addressing individual needs and societal imperatives, such as public health goals and legal justice. Ethical frameworks emphasise a victim-survivor-centred approach, prioritising respect, dignity and minimising harm while fulfilling legal and professional responsibilities. Integrating ethical principles into training for healthcare providers can support them in making nuanced decisions in complex cases.^5^

## Conclusion

Post-exposure prophylaxis plays a critical role in the comprehensive care of individuals who have experienced sexual assault. Timely intervention, including the administration of PEP and emergency contraception, is essential in reducing the risk of HIV, other STIs, Hepatitis B and unintended pregnancies. Healthcare providers must conduct thorough assessments, offer appropriate medical and psychosocial support and ensure effective follow-up care. Additionally, awareness and adherence to established guidelines and protocols, such as the use of the J88 form for evidence collection, are crucial for promoting justice and accountability in cases of sexual violence. By prioritising the health and wellbeing of victim-survivors, the healthcare community can contribute significantly to their recovery and empower them to reclaim their lives.
